# SNP by SNP by environment interaction network of alcoholism

**DOI:** 10.1186/s12918-017-0403-7

**Published:** 2017-03-14

**Authors:** Amin Zollanvari, Gil Alterovitz

**Affiliations:** 1 School of Engineering, Nazarbayev University, Astana, Kazakhstan; 2 Center for Biomedical Informatics, Harvard Medical School, Boston, MA USA; 3 Department of Electrical Engineering and Computer Science, Massachusetts Institute of Technology, Cambridge, MA USA

**Keywords:** GWAS, Alcoholism, SNP, Environment, Interaction, Network

## Abstract

**Background:**

Alcoholism has a strong genetic component. Twin studies have demonstrated the heritability of a large proportion of phenotypic variance of alcoholism ranging from 50–80%. The search for genetic variants associated with this complex behavior has epitomized sequence-based studies for nearly a decade. The limited success of genome-wide association studies (GWAS), possibly precipitated by the polygenic nature of complex traits and behaviors, however, has demonstrated the need for novel, multivariate models capable of quantitatively capturing *interactions* between a host of genetic variants and their association with non-genetic factors. In this regard, capturing the network of SNP by SNP or SNP by environment interactions has recently gained much interest.

**Results:**

Here, we assessed 3,776 individuals to construct a network capable of detecting and quantifying the interactions within and between plausible genetic and environmental factors of alcoholism. In this regard, we propose the use of first-order dependence tree of maximum weight as a potential statistical learning technique to delineate the pattern of dependencies underpinning such a complex trait. Using a predictive based analysis, we further rank the genes, demographic factors, biological pathways, and the interactions represented by our SNP $\times$SNP$\times$E network. The proposed framework is quite general and can be potentially applied to the study of other complex traits.

**Electronic supplementary material:**

The online version of this article (doi:10.1186/s12918-017-0403-7) contains supplementary material, which is available to authorized users.

## Background

Alcohol dependence is characterized by increasing tolerance to and consumption of alcohol, even in the face of adverse effects [1]. Almost 14% of alcohol consumers in the United States meet the criteria for alcohol dependence at some point in their lifetimes [2]. The consequences of alcohol dependence are severe. Overconsumption of alcohol is known to be a contributing factor to more than 60 diseases, including several types of cancer, and accounts for approximately 2.5 million deaths each year [3].

Alcoholism is very difficult to overcome once it initiates, and thus there has been much interest in preventing the onset of alcoholism altogether [3]. The construction of a genetic model of alcoholism has become increasingly possible with new genetic case–control studies of the disease [2]. Indeed, alcoholism is particularly amenable to a genetic model, as the genetic basis of the disease is strong. Adoption studies have demonstrated that children with alcoholic biological parents are likely to become alcoholics themselves, even if they are reared by adoptive parents in environments with few traces of alcohol [4]. Most adoption and twin studies suggest that 50–80% of variation in the phenotype is due to genetic factors [5]. That different people have different initial levels of tolerance to alcohol and thus different propensities to become physically addicted to it is further evidence of the genetic basis of the disease. That said, the same studies that have pointed to genetic factors have shown that demographic factors such as culture and level of education also contribute to alcoholism [6]. Thus, an effective model of alcoholism should incorporate both demographic and genetic information.

There have been several association studies that have sought to identify a small number of susceptibility loci for alcoholism [7]. However, complex traits like alcoholism are commonly underpinned by numerous factors, genetic as well as demographic, each of which has a small effect size [8]. Thus, many genome-wide association (GWA) studies on alcoholism have struggled to pinpoint individual single nucleotide polymorphisms (SNPs) that explain a good portion of the variation in the phenotype; the best odds ratios for individual SNPs reported in [2] were around 2, a relatively low figure. The detected variants with such a small effect size have explained a small portion of heritability. This problem is not only specific to alcoholism but to many other GWA studies commonly referred to as the “missing” heritability problem [9].

Various explanations have been suggested for the missing heritability [9], e.g., existence of rare variants with larger effect size that are not detectable with current genotyping techniques; more variants of small effect size that are not yet detected; and gene-gene (G×G) or gene-environment (G×E) interactions that are not discovered. The latter has resulted in various complementary studies to detect the SNP×SNP or SNP×E interactions in different phenotypes. For example, Jamshidi, et al. conducted a two-SNP interaction analysis and compared Cox’ regression models of pairs of SNPs with and without interaction term, i.e., SNP1+SNP2 vs. SNP1+SNP2+(SNP1×SNP2) [10]. For each pair of SNPs, the best model was selected based on the *p*-value of the likelihood ratio. Similarly, a logistic regression model SNP+E+(SNP×E) was used in [11] to identify a possible interaction between each SNP and the environment. The *p*-values of the interaction term was used to declare the significance of interaction. Limitations of linear or logistic regression analysis in detecting SNP-SNP interactions have been discussed elsewhere [12]. In particular, when the susceptibility to disease is caused by the interaction among several factors, the number of parameters required to fit a (logistic) regression model increases exponentially. This is not only computationally a challenge for constructing the regression model, but also this results in the quasi-complete separation effect (also known as “empty-cell” effect) in which case the estimate of parameters may not exist [13–15]. Therefore, rather than fitting one single unified regression model of many SNPs, researchers commonly fit many regression models of a pair of SNPs and either combine their results by further analysis (e.g. the gene-level analysis in [11]), or draw conclusion directly based on the results of the many fitted regression models (e.g., [10]).

Here, in an effort to discover plausible epistasis, i.e., non-additive SNPs association with alcoholism phenotype, we propose the use of first-order dependence tree of maximum weight. Although this technique has been proposed for the first time by Chow and Liu in [16], but its application in GWAS remains unexplored. This technique not only leads to an intuitive interpretation of detected interactions, but at the same time, provides the maximum likelihood estimate of the joint distribution of SNPs and/or environmental variables given the phenotypic label (case or control). At the core of this network approach is the mutual information of pairs of variables. However, in contrast with other network approaches such as [17–19] that also employ mutual information among SNPs/genes, the knowledge of joint distribution here creates a flow of information across nodes and edges of the network upon which inference is possible. In another words, the detected interactions are unified in a single probabilistic network. Based on the constructed network, we propose complementary analyses to rank the demographic factors, genes, biological pathways of alcoholism and compare our findings to prior domain knowledge.

## Results

### The SNP×SNP×E Network of Alcoholism

**Fig. 1:**
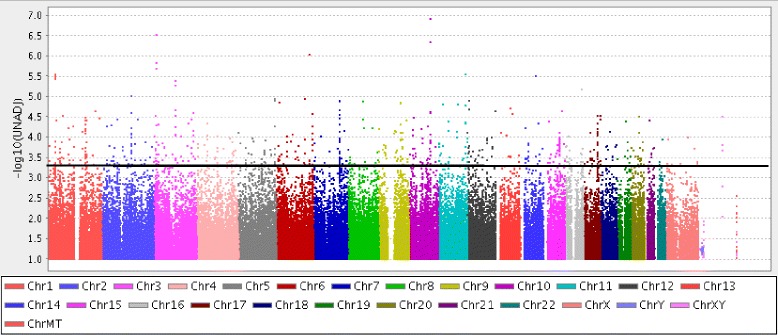
Manhattan plot of raw p-values from genome-wide association analysis (CMH test). Markers above the horizontal black line (*p*<0.0005) have been used in the iterative network construction. For the actual *p*-values and ranking of these 652 SNPs, see Additional file 2: Table S1

**Fig. 2:**
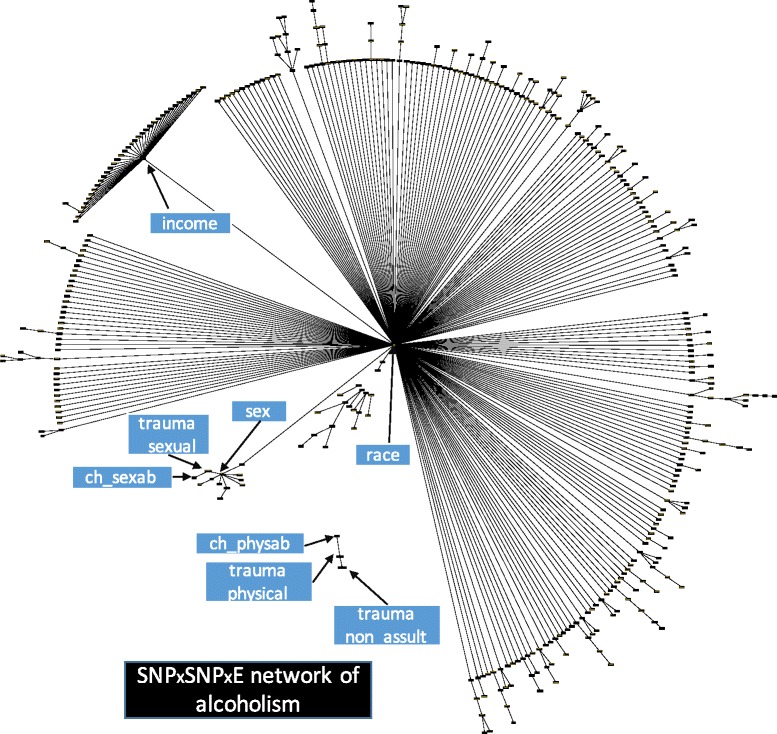
The SNP×SNP×E network of Alcoholism. The network contains 397 SNPs and 15 demographic variables. The nodes represent variables and an edge between two nodes represents their dependency quantified by conditional probabilities. For the node labels and the complete list of interactions see Additional file 3: Table S2. To enhance the quality of representation, we have removed the “alcoholism” node and the edges from this node to all other nodes

**Fig. 3:**
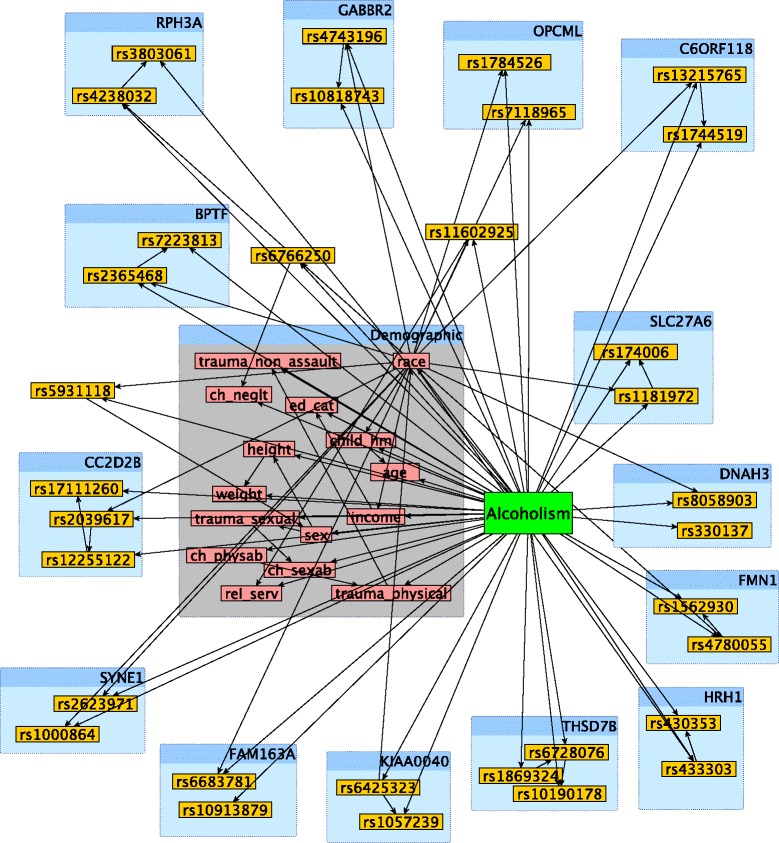
A subgraph of the SNP×SNP×E network in Fig. 2. All demographic factors are included, as well as the SNPs of several genes that have multiple SNPs in the network. Each blue box is labeled with the gene on which all of the SNPs within the box are found. The grey box contains all of the demographic factors

The Manhattan plot in Fig. 1 shows the significance of association of each SNP from genome-wide association analysis conducted in the available cohort of alcoholism. In this plot, each marker is represented by a dot and the –log_10_ (*p*-value) is displayed on the y-axis. Markers above the horizontal black line (*p*<0.0005) have been used in subsequent analysis for construction of the SNP×SNP×E network of alcoholism (see Methods Section for more details). Figures 2 and 3 provide the full picture of the SNP×SNP×E network and a sub-graph of this network, respectively. Data collection, preprocessing, and the working principle of the model are described in Methods Section. The network has 413 nodes (397 SNPs, 15 environmental factors (Table 1), and one phenotypic variable). An edge from a node (parent node) to another node (child node) indicates the conditional probability of the child node being in a state (homozygous wild-type or BB, heterozygotes or Bb, and homozygous mutant or bb) given the state of the parent node. Note that each node can have either a single parent or two parents, one of which is constantly the phenotypic node with two states (case and control). The 397 SNPs in the network are found in the 21 chromosomal regions that have been linked to alcoholism in previous association or linkage studies (all of which employed datasets and/or statistical methods different from ours).

**Table 1:** Demographic variables used in the SNP×SNP×E network

Variable	Level/Range
Income/yr $	0–10 K 10–20 K 20–30 K 30–40 K 40–50 K 50–75 K 75–100 K 100–150 K >150 K
Location of childhood home	large city/suburbs/small city/village/rural area
Level of education	less than H.S. grad/H.S. grad/some college/college grad or more
Gender	M/F
Age at interview	18–77
Race	AA/EA
Sexually abused as a child	yes/no
Otherwise physically abused as a child	yes/no
Neglected as a child	yes/no
Experienced sexual trauma	yes/no
Otherwise experienced physical trauma	yes/no
Experienced non-physical trauma	yes/no
Weight	85–435 lb
Frequency with which attends religious services	0–500
Height	49–80 in

Figures 2 and 3 confirm the frequent assertion that alcoholism is a byproduct of genetic and demographic factors. Based on Fig. 3, there seem to be a few likely reasons why such a synergy exists between demographic and genetic variables. First, the inclusion of race allowed the network to distinguish between SNPs that increase the risk of alcoholism only in African Americans (AAs) and those that do so only in European Americans (EAs). It is clear from Fig. 3 that there are a large number of SNPs that fit that description. As further evidence of race’s role, removal of race from the demographic-genetic classifier results in a decline in area under receiver operating characteristic curve (AUC) of 8.7%, the largest decline occurred by removing any feature from the network. Note that throughout this work, the AUC metric is merely used for ranking purposes (see Methods Section for details).

### Results of network composition analysis

We sought to rank the genes, demographic factors, biological pathways, and the interactions represented in our SNP$\times$SNP$\times$E network. In prior studies on modeling the gene effect based on SNP level interactions using regression analysis, the test statistic is obtained by summing the chi-squared 1° of freedom statistics within the gene, e.g., see [11]. However, here constructing an MWDT gives us an alternative and more intuitive way to combine the effect of various SNPs in a gene level analysis based on the AUC metric. In this regard, we sought to dissect our network to identify strong associations between alcoholism and genes, demographic variables, biological pathways, and interactions among factors. The results of the analysis (see Methods Section for details) are shown in Table 2. As described next, literature explicitly confirm some of the identified associations, providing further evidence that the network is not spurious. In other cases, we found evidence in the literature suggestive of the validity of associations. A few associations are not corroborated with the domain knowledge, but the general alignment of our results with prior work suggests that insight into the emergence of alcoholism. These associations are worthy candidates for further study.

**Table 2:** (a) The 14 most significant genes (*p* < 0.01) in the SNP×SNP×E network, including the intergenic set. 221 total genes were considered; (b) The four significant demographic factors (*p* < 0.05) in SNP×SNP×E network. 15 total demographic factors were considered; (c) The four significant interactions (*p* < 0.05) in the demographic-genetic model. 427 total interactions were considered

Table 2a	
Intronic/Intergenic SNPs	*p*-value
Intergenic	0.001
BLNK	0.002
BMPER	0.002
SERINC2	0.003
LGALS2	0.004
CPE	0.006
PDLIM5	0.006
PKNOX2	0.008
VEPH1	0.008
NPAS3	0.009
AMPD3	0.01
CADM3	0.01
DAB1	0.01
GLT25D2	0.01
Table 2b	
Demographic Factor	*p*-value
Race	0.001
Sex	0.001
Education Level	0.002
Income	0.002
Table 2c	
Factor-Factor Interaction	*p*-value
Race-Income	0.011
Sex-rs5933820	0.016
Race-rs8225	0.04
Income-Education Level	0.041

## Discussion

### Genes

Alcohol has a variety of effects on the body; many of these arise from alcohol’s activation of receptors in the brain [20]. A number of the genes identified in our analysis have important functions in the brain. In total, 9 of the 13 genes listed in Table 2a (excluding the intergenic set) either have been explicitly associated with alcoholism in the literature or have functional ties to the disease (e.g. are involved in brain activity). Three genes have been explicitly associated with the development of alcoholism. CPE has been identified in prior GWA studies on alcoholism [7], and it encodes the enzyme carboxypeptidase E, which activates neuropeptides [21], proteins crucial to communication among neurons. PKNOX2, which regulates the transcription of other genes and affects anatomical development [22], has been linked to various types of substance abuse in European women [23]. GLT25D2 was identified as related to alcoholism in a GWA study on a dataset that had no samples in common with ours [24]. Five other genes have functional ties to alcoholism and the development of the behavior (Additional file 1, Supplementary Notes, Section 1). While many identified genes were generally in alignment with prior knowledge, further work should be done to understand the associations between alcoholism and the five genes that went uncorroborated in the literature (BLNK, BMPER, PDLIM5, VEPH1, AMPD3). Finally, the high importance of intergenic SNPs in Table 2a is surprising, but similar SNPs have been tied in prior GWA studies to alcoholism [25], and the noncoding RNA that is transcribed from intergenic regions affects gene expression levels in some cases [26].

### G×G and G×E Interactions

Table 2b and c show demographic variables and interactions with a significant *p*-value (see Methods Section for details). Some of these factors and interactions are explicitly stated in prior studies. For example, a prior study [27] has demonstrated that alcohol consumption is negatively correlated with both income and educational status, both of which were deemed important demographic factors in Table 2b. The significance of the edge between income and education is sensible as well, as the conditional probability tables of the network indicate that a high level of education may be able to counteract a low level of income with respect to the development of alcoholism, and vice versa. Another prior study [28] provides the reason for the significance of the edge between race and income: there is a much stronger association between income and alcoholism in African Americans than in European Americans. Although no SNP-SNP interaction were deemed significant, the numerous SNP-SNP interactions that connect SNPs on the same gene (see Fig. 3) are reasonable, as SNPs that are closer together are more likely to interact and/or affect the same function [29].

There is also an interesting interaction between race and rs8225 in Table 2c (decline in AUC has *p*<0.04). While we used an AUC-metric-based approach to highlight this interaction, one may realize the importance of such link by examining the distribution of rs8225 among cases and controls in both races. As presented in Table 3, the distribution of this variant is substantially different between the two race groups in both cases and controls (difference of distribution of AAs and EAs in controls has a *p*<10^−15^ and in cases *p*<10^−15^ as determined by Cochran-Armitage test [30]). The within race group distribution of this variant is also significantly different between cases and controls (difference of distribution of AAs in controls and cases has a *p*<0.005 and this difference for EAs has *p*<0.0002 as determined by Cochran-Armitage test [30]). Another interesting interaction in Table 2c is the interaction of sex and rs5933820 (decline in AUC has p<0.02). While rs5933820 is located on the X chromosome, but its appearance as a significant interaction with gender in the context of alcoholism seems interesting and needs further validation and functional analysis.

**Table 3:** Distribution of rs8225 in the two race groups among cases and controls. The link between this variant and “race” group is determined to be statistically significant (see Table 2c)

Controls			
Race	2 C (wild type)	Heterozygous	2 T (variant)
African American	9.20%	41.80%	49.00%
European American	76.30%	22.20%	1.60%
Cases			
Race	2 C (wild type)	Heterozygous	2 T (variant)
African American	6.00%	37.5	56.50%
European American	70.40%	26.5	3.10%

### Biological Pathways

Twelve of the 14 biological pathways detected in our analysis (Table 4) have already been linked in the literature, either explicitly or indirectly, to the alcoholism. Two pathways have been explicitly cited for their involvement in the development of alcohol dependence. Fombonne, et al. demonstrated that children with long-term depression are at higher risk for alcohol dependence in adulthood [31]. The binding of GABA receptors, which are neuroactive ligand receptors, was found to be abnormally high in the brains of alcoholics [32]. Evidence in the literature suggests that four pathways may be involved in the emergence of alcoholism. It has been noted that alcohol inhibits the reorganization of the actin cytoskeleton [33]. Chronic exposure to alcohol reduces calcium signaling in response to glutamate receptor stimulation in neuronal cells [34]. Exposure of intestinal Gram negative bacteria to alcohol results in accumulation of acetaldehyde, which in turn increases tyrosine phosphorylation of adherens junction proteins [35]. Treatment of the ventral tegmental area in mice with glial cell line-derived neurotrophic factor activated the MAPK signaling pathway and reduced desire for alcohol [36]. Six pathways do not seem likely to be involved in the onset of alcoholism, but do appear to have links to the behavior (Additional file 1, Supplementary Notes, Section 1). Due to the overall alignment of the results of the analysis with the literature, it is likely that the two pathways that have not yet been explicitly tied in some way to alcoholism (dilated cardiomyopathy and hypertrophic cardiomyopathy) have links to the behavior; further study is required to confirm such associations.

**Table 4:** The 14 significant biological pathways (*p* < 0.05) in the demographic-genetic model. 186 total pathways were considered

KEGG Pathways	*p*-value
Calcium Signaling Pathway	0.001
Focal Adhesion	0.002
ECM Receptor Interaction	0.007
Arrhythmogenic Right Ventricular Cardiomyopathy (ARVC)	0.012
Hypertrophic Cardiomyopathy	0.012
Dilated Cardiomyopathy	0.012
Regulation of Actin Cytoskeleton	0.014
Oocyte Meiosis	0.014
Fc-Gamma Receptor-Mediated Phagocytosis	0.021
Long-term Depression	0.036
Adherens Junction	0.04
MAPK Signaling Pathway	0.04
Endocytosis	0.04
Neuroactive Ligand Receptor Interaction	0.047

## Conclusion

The analytical machinery proposed in this study can be potentially used to capture the complex multifactor effects between many genetic and environmental factors, providing a characterization of the underlying biological and environmental mechanism that determines the phenotype. The underlying framework is quite general and we anticipate seeing it applied to the study of other complex traits. The gene-gene-environment interactions are also known as one possible source of the “missing” heritability problem. In this regard, the next natural step is to use the proposed framework to quantify the proportion of the missing heritability explained by identified interactions.

## Methods

### Data Collection and Preprocessing

We utilized SAGE data [2], which featured 3,829 subjects and considered 948,658 SNPs from across the human genome, as well as several demographic variables. The data included human samples from three prior studies [2]; 30% of the individuals were African Americans and 70% were European Americans. The SAGE dataset includes 1,897 Diagnostic and Statistical Manual of Mental Disorders (DSM-IV) cases and 1,932 alcohol-exposed non-dependents. We used 15 environmental variables (demographic factors) that are listed in Table 1. Several demographic factors were left out, especially ones relating to comorbidities, because their distributions across the cases and controls were heavily imbalanced. All continuous demographic variables in the data (e.g. income) were discretized. We first removed any SNPs out of Hardy-Weinberg equilibrium (P < 0.0001). Hardy-Weinberg equilibrium tests were run separately on the African Americans and the European Americans in order to ensure identification of any SNPs common only in one race out of equilibrium. SNPs with minor allele frequency (MAF) below 0.01 or call rate below 98% were also removed from consideration, leaving a total of 934,128 SNPs. Finally, the 3,776 samples (1909 cases and 1867 controls) with a genotyping rate above 98% were maintained. A Cochran-Mantel-Haenszel (CMH) association test was used to rank the 934,128 SNPs [30]. The association analysis was performed with the software PLINK [37]. The top 652 SNPs (p < 0.0005) were maintained for network construction as detailed in the next few subsections.

### Maximum-weight Dependence Tree (MWDT)

First-order dependence tree of maximum weight is proposed initially by Chow and Liu [16] and further developed and evaluated by Friedman et al. [38]. Although there is no biological evidence that dependence between variables (genes or SNPs) follow a tree structure, but limitations on the number of available sample points compared to the complexity of the problem in hand require the joint distribution of variables be approximated by some simplifying assumptions. In this regard, tree dependence assumption is made to approximate a *n* ^*th*^ order joint probability distribution by a product of *n-1* s-order distributions. To understand the working principle in the context of GWAS, let *P*(**x**) denote the probability mass function of a random vector **x**. The mutual information between two variables (here SNP_1_ and SNP_2_) is given by

$$ I\left({\mathrm{SNP}}_1,{\mathrm{SNP}}_2\right)={\displaystyle \sum_{{\mathrm{SNP}}_1,{\mathrm{SNP}}_2}} P\left({\mathrm{SNP}}_1,{\mathrm{SNP}}_2\right)\  \log \left(\frac{P\left({\mathrm{SNP}}_1,{\mathrm{SNP}}_2\right)}{P\left({\mathrm{SNP}}_1\right) P\left({\mathrm{SNP}}_2\right)}\right) $$

Intuitively, *I*(SNP_1_, SNP_2_) measures the amount of information that SNP_1_ carries about SNP_2_ and vice versa. In a graphical representation of dependency among SNPs, we assume the dependencies have a tree structure (meaning each node has a single parent and one node (the root) has no parent), and assign to every edge of the tree an $I\left({\mathrm{SNP}}_i,{\mathrm{SNP}}_{m_i}\right)$. Then the tree with the maximum weight is the one that maximizes ${\displaystyle {\sum}_{i=1}^n I\left({\mathrm{SNP}}_i,{\mathrm{SNP}}_{m_i}\right)}$ where *m*_*i*_ denotes the parent node of node *i* and *n* is the number of SNPs under study. Note that there is no difficulty to maximize ${\displaystyle {\sum}_{i=1}^n I\left({\mathrm{SNP}}_i,{\mathrm{SNP}}_{m_i}\right)}$ without considering the class labels; however, doing so leads to a static network that may not differentiate one class from another. In other words, it is not possible to use the network as an inferential tool. The technique originally proposed in [16] resolves this problem by stratifying the samples at the outset and constructing one network for each class. Nevertheless, having a different network of interactions for each class will not only make the inference a more difficult and elusive task, but may not have a biological ground either.

In a case–control study, we can define a “class” variable C to measure the amount of information between SNPs given the phenotype (case or control). In this case, the maximum weight first-order dependence tree becomes the one with the maximum ${\displaystyle {\sum}_{i=1}^n I\left({\mathrm{SNP}}_i,{\mathrm{SNP}}_{m_i}\Big|\mathrm{C}\right)}$. By the first-order tree assumption on the structure of dependencies between SNPs, one can write the joint distribution between all SNPs given C as

$$ P\left({\mathrm{SNP}}_1,{\mathrm{SNP}}_2,\dots, {\mathrm{SNP}}_n\Big|\mathrm{C}\right)={\displaystyle \prod_{i=1}^n} P\left({\mathrm{SNP}}_i\Big|{\mathrm{SNP}}_{m_i},\mathrm{C}\right) $$

This decomposition of joint probability to product of “second-order” distributions or the distribution of first-order tree dependence leads to an algorithm that can “grow” the tree in polynomial time (Kruskal algorithm detailed in [16]). In practice, the knowledge of conditional probability distributions is not available, and they must be estimated from data. Nevertheless, it can be shown that due to decomposition of joint probability distributions as mentioned above, the strategy that finds the tree with maximum weights is also the maximum likelihood estimate (MLE) of the joint distribution. In other words, finding the tree with maximum ${\displaystyle {\sum}_{i=1}^n\widehat{I}\left({\mathrm{SNP}}_i,{\mathrm{SNP}}_{m_i}\Big|\mathrm{C}\right)}$, with $\widehat{I}\left({\mathrm{SNP}}_i,{\mathrm{SNP}}_{m_i}\Big|\mathrm{C}\right)$ being the sample estimate of $I\left({\mathrm{SNP}}_i,{\mathrm{SNP}}_{m_i}\Big|\mathrm{C}\right)$, is equivalent to the MLE of the joint distribution of SNPs, *P*(SNP_1_, SNP_2_, …, SNP_*n*_|C), under the first order dependence tree structure (see [16]). This implies that if the true dependence between SNPs has a tree structure, then as the sample size increases, the estimated trees converge to the true tree with probability one. For further details on estimating $I\left({\mathrm{SNP}}_i,{\mathrm{SNP}}_{m_i}\Big|\mathrm{C}\right)$, see Additional file 1, Supplementary Notes, Section 2. Another interesting feature of MWDT is that approximating and estimating the joint distribution of SNPs create a flow of information among nodes of the network. As opposed to other network approaches based on mutual information [17–19], this interesting property of the network gives us the ability to employ the network as an inferential tool. For example, for an observation of unknown class, one can assign a case label if

$$ {\displaystyle \prod_{i=1}^n} P\left({\mathrm{SNP}}_i\Big|{\mathrm{SNP}}_{m_i},\mathrm{C}=\mathrm{case}\right) > {\displaystyle \prod_{i=1}^n}\left({\mathrm{SNP}}_i\Big|{\mathrm{SNP}}_{m_i},\mathrm{C}=\mathrm{control}\right) $$

### AUC in Ranking Networks of Interactions

From the previous section, we have to note that the MWDT guarantees the maximum likelihood estimate of the joint distribution given the true tree dependency among a set of given SNPs. However, for a set of SNPs of size *n* (here 652 SNPs selected as described before), there will be 2^*n*^-1 potential maximum weight networks that can be constructed on any subset of *n* variables. Of course one may choose to grow the tree on all *n* SNPs but here we propose a complementary step to further narrow down the list of potential genetic factors used in the proposed network of alcoholism. To do so, we use the network as a classifier and use the AUC to rank a set of potential networks (see next subsection) and choose the one with the highest AUC. Unless otherwise stated, we employ 3-fold cross-validation procedure to compute AUCs. Nevertheless, since for the initial dimensionality reduction step, we use the CMH test on the full training data, we shall not interpret AUC as the predictive ability of our constructed network on a subset of SNPs and/or other factor. In other words, the use of AUC here is merely a measure to rank constructed sub-networks of interactions.

### Ranking mechanism

To construct the optimal network of interactions, two approaches were employed: one is a backward sequential iterative approach described below, and the other is an approach based on a combination of linkage disequilibrium (LD) analysis [39] and the backward iterative approach. In the (backward) iterative approach, the MWDT was first trained with the remaining SNPs and the 15 demographic variables as part of the network. In each subsequent iteration, the 50 SNPs with the largest CMH *p*-values were removed and a new network was constructed using the reduced list of SNPs. The best network was the one with the highest AUC in differentiating cases from controls. The LD analysis-based approach sought to eliminate redundant SNPs. LD analysis was performed and SNPs that were strongly linked (i.e. frequently co-occurred in both the cases and the controls) were grouped into bins. The approach outlined by Carlson, et al. [40], with the r^2^ threshold lowered from 0.8 to 0.4, was used to produce a single tag SNP for each LD bin. Only the tag SNPs were maintained, and the iterative approach was applied to them. This approach ensures that multiple SNPs that are proxies due to low LD distance are not selected. The tag SNP acts as a proxy for all SNPs in that region. The best networks from the two approaches were compared, and the one with the highest AUC was selected as the SNP×SNP×E network.

### Analysis of network composition

To study the gene level interactions with the phenotype based on SNP level variations, we enumerate all genes with at least one SNP in the network. For each gene, we construct a sub-network of SNPs involved in the full SNP×SNP×E network located on that gene and record the AUC of a newly constructed sub-network. We consider race and sex as part of each sub-network. This would unlock the full potential of race- or sex-specific SNPs. In a sense, this analysis is similar to the adjustment for sex and age in the classical regression analysis.

We next considered important demographic features. To evaluate the importance of each demographic factor, we calculated the decline in resubstitution AUC (AUC on the training set) upon removal of that factor and all edges connected to it from the full SNP×SNP×E network. Resubstitution was used because the response of cross-validation AUC to minor changes is relatively imprecise due to larger variance of cross-validation estimators [41]. We used the Molecular Signatures Database [42] to determine the lists of genes related to 186 pathways from the Kyoto Encyclopedia of Genes and Genomes (KEGG) [43]. For each KEGG pathway, we recorded the AUC of the corresponding sub-network constructed using SNPs in the full network that are within the pathways’s genes, as well as race and sex. Finally, to detect most important interactions, we successively removed each edge in the full SNP×SNP×E network and recorded the decline in AUC. The analysis left us with an AUC for each gene, pathway, and a decline in AUC for each demographic feature and interaction. Rather than reporting the actual AUCs, which here is merely used for ranking purposes, we calculated a *p*-value associated with each AUC. Although here ranking based on AUC or *p*-value leads to the same result, we use the *p*-value threshold of 0.05 (non-adjusted) to narrow down the list.

To determine a *p*-value for each gene- or pathway-specific network, we constructed 1,000 networks, each with the same number of nodes for which the AUC in question was calculated, and determined their AUCs. The set of genetic features for each of the 1,000 networks was drawn randomly from the background set of SNPs. Race and sex were included as features in all 1,000 networks in order to ensure parity with the procedure used to generate the gene- or pathway-specific network. The list of 1,000 random AUCs enabled the calculation of a *p*-value for the AUC in question.

To determine the statistical significance of each decline in AUC (used for quantifying the importance of each demographic variable and the interactions in the SNP×SNP×E network), we used the same background set to construct 1,000 random networks with the same set of demographic factors and the same number of SNPs as in the SNP×SNP×E network. For each randomly generated model, we recorded the decline in AUC upon removal of a random SNP (in the case of the declines in AUC for demographic factors) or a random edge (in the case of the declines in AUC for interaction). The 1,000 random declines in AUC enabled the calculation of a *p*-value for the decline in AUC of interest. Each gene, demographic factor, pathway, and interaction relationship was now associated with a *p*-value.

## Additional files

Additional file 1: Supplementary Notes: The first section in this file provides the evidence for functional ties between some of the implicated genes/pathways and alcoholism. The second section in this file details the maximum likelihood estimate of the conditional mutual information. (DOCX 170 kb)

Additional file 2: **Table S1.** This file provides the list of SNPs with CMH test p-value < 0.0005. (XLSX 52 kb)

Additional file 3: **Table S2.** This file provides the complete list of interactions in the SNPxSNPxE network. (XLSX 56 kb)

## Abbreviations

AA: African Americans
AUC: Area under receiver operating characteristic curve
CMH: Cochran-Mantel-Haenszel
EA: European Americans
GWA: genome-wide association
KEGG: Kyoto Encyclopedia of Genes and Genomes
LD: Linkage disequilibrium
MAF: Minor allele frequency
MLE: Maximum likelihood estimate
MWDT: Maximum-weight Dependence Tree
SNP: Single nucleotide polymorphisms
