# Supplementary material for: SNP by SNP by environment interaction network of alcoholism
Source: BMC Syst Biol. 2017 Mar 14;11(Suppl 3):19. doi: 10.1186/s12918-017-0403-7 (PMC5374593; doi:10.1186/s12918-017-0403-7)
Supplement: Supplementary file 3 — Table S2. This file provides the complete list of interactions in the SNPxSNPxE network. (XLSX 56 kb) [file 12918_2017_403_MOESM3_ESM.xlsx]

**SNP by SNP by Environment Interaction Network of Alcoholism**

**Supplementary Information**

Amin Zollanvari^1,2^; Gil Alterovitz^2,3^

**^1^School of Engineering, Nazarbayev University, Astana, Kazakhstan; ^2^Center for Biomedical Informatics, Harvard Medical School, Boston, MA; ^3^Department of Electrical Engineering and Computer Science, Massachusetts Institute of Technology, Cambridge, MA**

**Section 1:**

**Genes Suggested in the Literature.** SERINC2 was found to be differentially expressed (with a false discovery rate below 0.005) when HepG2 (liver) cells were exposed to ethanol [1], the type of alcohol in drinks. LGALS2 was differentially expressed (more than a two-fold change) between the brains of two populations of mice with different levels of ethanol sensitivity [2]. NPAS3 encodes a transcription factor that is part of the basic helix-loop- helix (bHLH) family of transcription factors, some of which are known to regulate toxin metabolism [3]. In addition, a study on mice demonstrated that NPAS3 is expressed in interneurons, and that deficiencies in the NPAS3 transcription factor are associated with psychosis [4]. A study on larval zebrafish indicated that the CADM3 protein plays a role in the development of axons [5]. Mutations in DAB1 in developing mice have altered the positions of neurons in the cerebral cortex [6]. Other work has provided evidence of an association between alcoholism and DRD4, which encodes the dopamine D4 receptor, present in the central nervous system [7]. TAF1A was found to be differentially expressed (more than a two-fold change) in the livers of mice exposed to ethanol [8].

**Genes with Multiple SNPs.** Since SNPs in LD were pruned prior to construction of the network, it is unlikely that LD accounted for the large number of genes with multiple SNPs in the model (see Figure 2 and 3 in the paper). Thus, strong associations between such genes and alcoholism might be expected, even though no such gene was deemed statistically significant. Indeed, the literature provides plenty of evidence in favor of such associations. Several explicit associations were found. One gene with multiple SNPs in the model, GABBR2, encodes the GABA_B2_ receptor, agonists of which have reduced addiction to alcohol in rats [9]. Other such genes, including CC2D2B, BPTF, and HRH1, have been cited as related to alcoholism in GWA studies [1] that used datasets or association analyses different from ours. Some indirect associations were found as well. For example, a paralog of THSD7B, another gene with multiple SNPs in the model, encodes a protein that was found in the neural tubes of zebrafish embryos [10]. RPH3A encodes the protein rabphilin, which affects synaptic vesicles, the carriers of neurotransmitters [11]. Thus, both genes with multiple SNPs in the model and those identified as statistically significant are promising candidates for further study with regard to the etiology of alcoholism.

**Additional Linked Biological Pathways.** Both alcoholism and arrhythmogenic right ventricular cardiomyopathy cause infiltration of the right ventricular myocardium by fatty tissue [12]. During hepatic fibrogenesis, a common consequence of alcohol abuse, hepatic stellate cells are activated; such cells have receptors for extracellular matrix (ECM) components [13]. The resultant binding of such cells to the ECM activates focal adhesion kinase (FAK), which aids in the maintenance of focal adhesions [13]. Furthermore, exposure to alcohol in female mice reduced the quality of oocytes and disrupted chromosome segregation in ooctye meiosis. Alcoholic cirrhosis increases the incidence of pyogenic infections, which reduce the effectiveness of Fc-gamma receptor-mediated phagocytosis, a type of endocytosis [14].

**Section 2**

**Maximum Likelihood Estimate of Conditional Mutual Information**

The sample estimate of the conditional mutual information, $\hat{I}(\mathrm{SNP}_{i},\mathrm{SNP}_{m_{i}}|C)$, is given by

$$\hat{I}\left( \mathrm{SNP}_{i},\mathrm{SNP}_{m_{i}} | C \right)=\sum_{\mathrm{SNP}_{i},\mathrm{SNP}_{m_{i}}} f\left( \mathrm{SNP}_{i},\mathrm{SNP}_{m_{i}}|C \right) log(\frac{f(\mathrm{SNP}_{i},\mathrm{SNP}_{m_{i}}|C)}{f(\mathrm{SNP}_{i}|C)f(\mathrm{SNP}_{m_{i}}|C)})$$

where $f\left( \mathrm{SNP}_{i},\mathrm{SNP}_{m_{i}}|C \right)$and $f\left( \mathrm{SNP}_{i} | C \right)$ are the MLE of the joint distribution of $P\left( \mathrm{SNP}_{i},\mathrm{SNP}_{m_{i}}|C \right)$ and $P\left( \mathrm{SNP}_{i}|C \right)$, respectively, given by

$$f\left( \mathrm{SNP}_{i},\mathrm{SNP}_{m_{i}}|C \right)=\frac{\#\{\mathrm{SNP}_{i},\mathrm{SNP}_{m_{i}}|C\}}{\sum_{\mathrm{SNP}_{i}, \mathrm{SNP}_{m_{i}}\in\left( BB, Bb, bb \right)} \#\{\mathrm{SNP}_{i},\mathrm{SNP}_{m_{i}}|C\}}$$

and

$$f\left( \mathrm{SNP}_{i}|C \right)=\frac{\#\{\mathrm{SNP}_{i}|C\}}{\sum_{\mathrm{SNP}_{i}\in\left( BB, Bb, bb \right)} \#\{\mathrm{SNP}_{i}|C\}}$$

with $\#\{\mathrm{SNP}_{i},\mathrm{SNP}_{m_{i}}|C\}$ being the number of sample points in group C (case/control) such that the *i*^th^ SNP and its parent take one of the possible genotypes BB, Bb, or bb. Note that we have an abuse of notation here as for denoting the *i*^th^ SNP and the genotype under study at this locus, we use $\mathrm{SNP}_{i}$.

**References**

[1] Pochareddy, S. (2010). *Transcriptional Regulation of the Human Alcohol Dehydrogenases and Alcoholism.* Ph.D. Thesis. Indiana University: U.S.

[2] MacLaren, E. J.,  *et al.* Cerebellar Gene Expression Profiling and eQTL Analysis in Inbred Mouse Strains Selected for Ethanol Sensitivity. *Alcohol Clin Exp Res* 29 (2005): 1568-79.

[3] Brunskill, E. W.,  *et al.* Characterization of Npas3, a novel basic helix-loop-helix PAS gene expressed in the developing mouse nervous system. *Mechanisms of Development* 88 (1999): 237-241.

[4] Erbel-Sieler, C.,  *et al.* Behavioral and regulatory abnormalities in mice deficient in the NPAS1 and NPAS3 transcription factors. *Proc Natl Acad Sci USA* 101 (2004): 13648-13653.

[5] Hunter, P. R.,  *et al.* Localization of Cadm2a and Cadm3 proteins during development of the zebrafish nervous system. *J Comp Neurol* 519 (2011): 2252-70.

[6] Ohshima, T.,  *et al.* Synergistic contributions of cyclin-dependant kinase 5/p35 and Reelin/Dab1 to the positioning of cortical neurons in the developing mouse brain. *Proc Natl Acad Sci USA* 98 (2001): 2764-2769.

[7] Muramatsu, T.,  *et al.* Association between alcoholism and the dopamine D4 receptor gene. *J Med Genet* 33 (1996): 113-115.

[8] Yin, H.,  *et al.* Differential gene expression and lipid metabolism in fatty liver induced by acute ethanol treatment in mice. *Toxicology and Applied Pharmacology* 223 (2007): 225-233.

[9] Liang, J.,  *et al.* The GABAB receptor allosteric modulator CGP7930, like baclofen, reduces operant self-administration of ethanol in alcohol-preferring rats. *Neuropharmacology* 50 (2006): 632-639.

[10] Wang, C.,  *et al.* Zebrafish Thsd7a is a neural protein required for angiogenic patterning during development. *Developmental Dynamics* 240 (2011): 1412-1421.

[11] Deak, F.,  *et al.* Rabphilin regulates SNARE-dependent re-priming of synaptic vesicles for fusion. *EMBO J* 25 (2006): 2856-2866.

[12] Britton, R. S.,  *et al.* Intracellular Signaling Pathways in Stellate Cell Activation. *Alcohol Clin Exp Res* 23 (1999): 922-5.

[13] Cebral, E.,  *et al.* Deleterious effects of chronic moderate alcohol intake by female mice on preimplanation embyro growth in vitro. *Alcohol and Alcoholism* 34 (1999): 551-8.

[14] Schiff, D. E.,  *et al.* Increased Phagocyte Fc-gammaRI Expression and Improved Fc-gamma Receptor-Mediated Phagocytosis After In Vivo Recombinant Human Interferon-gamma Treatment of Normal Human Subjects. *Blood* 90 (1997): 3187-3194.
